# Well-to-wake analysis of ethanol-to-jet and sugar-to-jet pathways

**DOI:** 10.1186/s13068-017-0698-z

**Published:** 2017-01-24

**Authors:** Jeongwoo Han, Ling Tao, Michael Wang

**Affiliations:** 1 Systems Assessment Group, Energy Systems Division, Argonne National Laboratory, 9700 S. Cass Avenue, Argonne, IL 60439 USA; 2 National Renewable Energy Laboratory, 15013 Denver West Parkway, Golden, CO 80401 USA

**Keywords:** Life-cycle analysis, Well-to-wake analysis, Ethanol-to-jet, Sugar-to-jet, Greenhouse gas emissions, Fossil fuel use, Water consumption

## Abstract

**Background:**

To reduce the environmental impacts of the aviation sector as air traffic grows steadily, the aviation industry has paid increasing attention to bio-based alternative jet fuels (AJFs), which may provide lower life-cycle petroleum consumption and greenhouse gas (GHG) emissions than petroleum jet fuel. This study presents well-to-wake (WTWa) results for four emerging AJFs: ethanol-to-jet (ETJ) from corn and corn stover, and sugar-to-jet (STJ) from corn stover via both biological and catalytic conversion. For the ETJ pathways, two plant designs were examined: integrated (processing corn or corn stover as feedstock) and distributed (processing ethanol as feedstock). Also, three H_2_ options for STJ via catalytic conversion are investigated: external H_2_ from natural gas (NG) steam methane reforming (SMR), in situ H_2_, and H_2_ from biomass gasification.

**Results:**

Results demonstrate that the feedstock is a key factor in the WTWa GHG emissions of ETJ: corn- and corn stover-based ETJ are estimated to produce WTWa GHG emissions that are 16 and 73%, respectively, less than those of petroleum jet. As for the STJ pathways, this study shows that STJ via biological conversion could generate WTWa GHG emissions 59% below those of petroleum jet. STJ via catalytic conversion could reduce the WTWa GHG emissions by 28% with H_2_ from NG SMR or 71% with H_2_ from biomass gasification than those of petroleum jet. This study also examines the impacts of co-product handling methods, and shows that the WTWa GHG emissions of corn stover-based ETJ, when estimated with a displacement method, are lower by 11 g CO_2_e/MJ than those estimated with an energy allocation method.

**Conclusion:**

Corn- and corn stover-based ETJ as well as corn stover-based STJ show potentials to reduce WTWa GHG emissions compared to petroleum jet. Particularly, WTWa GHG emissions of STJ via catalytic conversion depend highly on the hydrogen source. On the other hand, ETJ offers unique opportunities to exploit extensive existing corn ethanol plants and infrastructure, and to provide a boost to staggering ethanol demand, which is largely being used as gasoline blendstock.

**Electronic supplementary material:**

The online version of this article (doi:10.1186/s13068-017-0698-z) contains supplementary material, which is available to authorized users.

## Background

Jet fuel consumption in the US has been estimated at 3.0 trillion MJ in 2015, accounting for 10.1% of energy supplied to the US transportation sector, and this consumption is projected to steadily increase to 3.7 trillion MJ in 2040 [1]. Greenhouse gas (GHG) emissions from jet fuel combustion in the US were 149 million ton CO_2_e in 2014, accounting for 8.5% of total GHG emissions by the US transportation sector [2]. Globally, jet fuel consumption has been estimated at 377 billion liters or 13.1 trillion MJ in 2012 [3]. Moreover, air traffic is expected to grow steadily: the US Energy Information Administration projected revenue passenger miles in the US will increase from 4.0 trillion miles in 2015 to 9.6 trillion miles in 2040 [1]. In response to growing environmental concerns, the aviation industry is exploring environmentally, economically, and socially sustainable solutions to reduce fuel consumption and GHG emissions for the sustainable growth of air traffic [4]. While fuel consumption can be reduced by the development and use of more efficient aircraft, shorter routing, and optimized flight management and planning, it is also beneficial to displace fossil jet fuels with low-carbon bio-based jet fuels to reduce GHG emissions significantly.

To promote bio-based jet fuel deployment, several organizations (e.g., the US Federal Aviation Administration, the US Air Force, the US Navy, the International Civil Aviation Organization, and the European Union) have committed to using bio-based jet fuels. For example, the US Department of Defense purchased about 7.6 million liters of alternative fuels between fiscal years 2007 and 2014 for testing purposes [5]. The purchased alternative fuels include largely renewable jet and diesel from hydroprocessed ester and fatty acids (HEFA) and Fischer–Tropsch jet (FTJ) along with a smaller volume of alcohol-to-jet (ATJ), synthetic iso-paraffins produced via direct sugar-to-hydrocarbon technology, and Fischer–Tropsch diesel [6]. Renewable Jet from HEFA, also known as hydroprocessed renewable jet (HRJ), is produced through hydroprocessing of fatty acids from hydrogenation of vegetable, algae, or waste oil, while FTJ is produced from gasification of natural gas (NG), coal, and biomass and with a subsequent Fischer–Tropsch synthesis. In the current ATJ process, alcohol (e.g., ethanol, methanol, or iso- or normal-butanol) is first dehydrated and converted into linear olefins via catalytic oligomerization. Then, the olefinic double-bonds are saturated via a hydrotreating process to make ATJ. For commercial aviation uses, the American Society for Testing and Materials (ASTM) International has certified HRJ, FTJ (such as Fischer–Tropsch synthetic paraffinic kerosene and Fischer–Tropsch synthetic kerosene with aromatics), synthetic iso-paraffins produced via direct sugar-to-hydrocarbon, and butanol-to-jet technologies. Other production pathways undergoing certification processes include other ATJ pathways, pyrolysis-based hydrotreated depolymerized cellulosic jet, other sugar-to-jet (STJ) pathways, and catalytic hydrothermolysis jet [7].

The key advantages of the alternative jet fuels (AJFs) over petroleum jet fuel are potential reductions in petroleum consumption and GHG emissions, which need to be evaluated on a life-cycle basis. Several life-cycle analyses of AJFs have been published. Using HEFA production details provided by UOP, Shonnard et al. [8] and Fan et al. [9] estimated the well-to-wake (WTWa) GHG emissions associated with camelina- and pennycress-based HRJ using an energy-based allocation method, with results of 22 and 33 g CO_2_e/MJ, respectively. These studies assumed little land use change (LUC) impact of these fuels because the feedstocks are rotational crops. Ukaew et al. [10] investigated soil organic carbon impacts of rapeseed cultivated in inter-year rotation with wheat (wheat–wheat-rapeseed rotation) as compared to the reference wheat–wheat-fallow rotation. They modeled the top five wheat-producing counties in ten different states in the US, and demonstrated large variations in soil organic carbon changes (−0.22 to 0.32 Mg C/ha/year) incurred by rapeseed cultivation in rotation with wheat, depending on location and farming practices. The soil organic carbon changes resulted in direct LUC impacts estimated to range from −43 to 31 g CO_2_e/MJ HRJ. Ukaew et al. [11] further examined the impact of crop prices on LUC estimates for HRJ from canola produced in North Dakota, and showed a strong correlation between canola price and LUC. Bailis and Baka [12] estimated WTWa GHG emissions from jatropha-based HRJ to be 40 g CO2e/MJ without LUC, and estimated that direct LUC GHG emissions would range from −27 to 101 g CO_2_e/MJ, depending on the soil type. In addition, Seber et al. [13] discussed the GHG emissions from waste oil- and tallow-based HRJ, which depend highly on the system boundary for the waste feedstock. Other studies examined the GHG emissions of HRJ from camelina, algae, and jatropha with various farming and fuel production assumptions [14, 15]. Hydrothermal liquefaction, using algae as the feedstock, has also been examined for AJF production [16, 17]. On the other hand, Skone and Harrison [18] investigated FTJ production from coal and biomass using a process engineering model. The study estimated the FTJ’s WTWa GHG emissions to range from 55 to 98 g CO_2_e/MJ, depending on biomass type and share, catalyst type, carbon management strategy, and co-product handling method. Lastly, the GHG emissions associated with jet fuel obtained from mallee via pyrolysis was estimated at 49 g CO_2_e/MJ [19].

Since these studies were conducted with different assumptions and life-cycle analysis (LCA) approaches, efforts were made to compare these different AJFs on a consistent basis. Stratton et al. [20] compared the GHG emissions associated with FTJ from NG, coal, and biomass and HRJ from several oil crops and algae with those from petroleum jet fuel. They showed that FTJ from biomass and HRJ from vegetable oil and algae have potentials to reduce GHG emissions up to 102 and 66%, respectively, relative to petroleum jet depending on process assumptions and LUC emissions. These authors further discussed the impact of variation in several parameters and key LCA issues (e.g., co-product handling method and LUC) on the GHG emissions of FTJ and HRJ [21]. Elgowainy et al. [22] expanded the AJF options by adding pyrolysis jet fuel derived from corn stover, and updated key parameters for FTJ and HRJ as well as petroleum jet fuel. Han et al. [23] refined HRJ production process assumptions on the basis of fatty acid profiles of oil seeds, and showed that WTWa GHG emissions can be reduced by 41–63% (for HRJ), 68–76% (for pyrolysis jet fuel), and 89% (for FTJ from corn stover) relative to petroleum jet fuel. Agusdinata et al. [24] conducted WTWa analyses of bio-based jet fuel from non-food crops (e.g., camelina, algae, corn stover, switchgrass, and woody biomass), and projected a substantial GHG emissions reduction in 2050 under several economic and policy assumptions.

Compared to HRJ and FTJ, only a few WTWa studies on ATJ and STJ are available as summarized in Table 1. Cox et al. [25] evaluated the STJ from sugarcane molasses, and estimated its GHG emissions at 80 g CO_2_e/MJ, using a system expansion method. On the other hand, Moreira et al. [26] estimated the GHG emissions of STJ from sugarcane at 8.5 g CO_2_e/MJ, using a system expansion method. The large difference in the GHG emissions between these two studies stemmed from differing approaches to estimating indirect effects. Cox et al. [25] assumed that sorghum production will increase as sugarcane is used as a jet fuel feedstock, resulting in LUC GHG emissions of over 100 g CO_2_e/MJ from the increased sorghum production. Moreira et al. [26], on the other hand, used the Global Trade Analysis Project model to estimate the LUC, and reported subsequent LUC GHG emissions of 12 g CO_2_e/MJ. Staples et al. [27] examined nine advanced fermentation pathways from sugarcane, corn, and switchgrass (including both ATJ and STJ), and showed that the WTWa GHG emissions of jet fuels from these three feedstocks varied significantly depending on the feedstock-to-fuel conversion routes and the co-product handling method: −27 to 20 g CO_2_e/MJ for sugarcane, 48 to 118 g CO_2_e/MJ for corn, and 12 to 90 g CO_2_e/MJ for switchgrass without LUC. Additionally, they investigated the direct LUC effects for three cases (low, baseline, and high emissions), and reported estimated LUC GHG results of 20–47 g CO_2_e/MJ for sugarcane, 38–101 g CO_2_e/MJ for corn, and 1–12 g CO_2_e/MJ for switchgrass. Recently, Budsberg et al. [28] examined the WTWa GHG emissions and fossil fuel use of ATJ from poplar. They investigated two options for H_2_ production: NG steam methane reforming and lignin gasification resulted in 60–66 and 32–73 gCO_2_e/MJ, respectively.

**Table 1:** WTWa GHG emissions of STJs and ATJs from previous studies (numbers in the parenthesis indicates estimated ranges)

References	Feedstock	Co-products	Co-product handling methods	WTWa GHG emissions (g CO_2_e/MJ)	Note
Cox et al. [25]	Sugarcane	Sugar, electricity, steam	Displacement	80	Including indirect impact from increased sorghum production
			Market value allocation	22	
Moreira et al. [26]	Sugarcane	Electricity, yeast^e^	Displacement	8.5	With LUC emissions^a^ (12 g CO_2_e/MJ)
Staples et al. [27]	Sugarcane	Electricity	Displacement	−4.9 (−27 to 2.1)	Without LUC^b^
			Market value allocation	12.7 (6.8 to 19.7)	
	Corn	Distiller dry grains with solubles	Displacement	65.6 (50.1 to 117.4)	Without LUC^c^
			Market value allocation	62.6 (47.6–117.5)	
	Switchgrass	Electricity	Displacement	37.4 (11.7 to 89.8)	Without LUC^d^
			Market value allocation	37.4 (17.3 to 89.8)	
Budsberg et al. [28]	Poplar	Electricity	Displacement	60 to 66	Without LUC; H_2_ from NG SMR
			Displacement	32 to 73	Without LUC; H_2_ from biomass gasification

^a^LUC GHG was estimated at 12 g CO_2_e/MJ

^b^LUC GHG was estimated at 20–47 g CO_2_e/MJ

^c^LUC GHG was estimated at 38–101 g CO_2_e/MJ

^d^LUC GHG was estimated at 1–12 g CO_2_e/MJ

^e^The jet production process used in Moreira et al. [26] recovers and export yeast as a co-product

Cox et al. [25] and Moreira et al. [26], however, examined only STJ produced via biological conversion from sugarcane, which is not widely available for fuel production outside Brazil. Staples et al. [27] included corn and corn stover, which are more relevant to the US biofuel industry. However, Staples et al. [27] divided the production process into four stages (pretreatment, fermentation, extraction, and upgrading), and employed process assumptions for each stage (such as efficiency, energy, and mass balances) from various literature sources to estimate energy consumption in each fuel production route rather than developing a conversion process as an integrated plant. Also, the efficiencies and process energy requirements of certain processes (such as fermentation and ETJ processes) were based on theoretical maximum and expert opinions while other processes (e.g., pretreatment) were from previous techno-economic analyses (TEA) of other biofuel production (such as ethanol). Thus, assumptions (e.g., plant scale) might be inconsistent among stages and processes that might not be well-integrated. Moreover, STJ produced via catalytic conversion is yet to be investigated.

To conduct WTWa analysis on emerging ATJ and STJ from the feedstocks relevant to the US using well-integrated process assumptions, the present study incorporated the results from three TEAs into the Greenhouse gases, Regulated Emissions and Energy use in Transportation (GREET^®^) model and systematically estimated WTWa GHG emissions reductions as well as fossil fuel use and water consumption by the use of these new AJFs relative to petroleum jet fuel [29]. The three TEA studies include ethanol-to-jet (ETJ) production [30], STJ production via biological conversion [31], and STJ via catalytic conversion [32]. Note that ETJ is a subset of ATJ processes using ethanol as an intermediate. Key advantages of ETJ pathways over other ATJ or alternative fuel pathways include the large feedstock availability (both sugar/starch and lignocellulosic biomass) and the technological maturity of fuel ethanol conversion, especially with starch and sugar feedstocks. Currently in the US, ethanol is largely used as a fuel additive in E10 gasoline. The Renewable Fuels Association estimated the US ethanol production at 55.6 billion liters in 2015, while the US gasoline consumption was 553 billion liters in 2015 and is expected to be reduced in the future [1, 33]. Thus, with the 10% “blend wall,” ethanol production could potentially surpass consumption in the US E10 market, which would create opportunities for ETJ pathways.

This study presents the baseline LCA results of corn-based ETJ (using integrated and distributed plants), corn stover-based ETJ (using integrated and distributed plants), and corn stover-based STJ (via biological and catalytic conversions) as compared to conventional petroleum jet using the GREET model. The GREET model is an attributional LCA model while LUC impacts are estimated via a consequential analysis. The STJ pathway via catalytic conversion uses H_2_ from external source. After describing the baseline results, we assess the key drivers for the GHG reductions through sensitivity analyses that examine the influence of the following: ethanol production pathways for ETJ with a distributed ETJ production, H_2_ sources for STJ produced via catalytic conversion, and co-product handling methods. Also, sensitivity analyses on key parametric assumptions are provided to show the impact of these parameters on the WTWa results. Lastly, GHG emissions for different jet fuel production pathways using one metric ton of corn stover as a uniform feedstock are presented to examine the impact of liquid fuel yields and GHG intensities of AJFs on the total GHG emissions.

## Methods

### WTWa analysis system boundary and methods

**Fig. 1:**
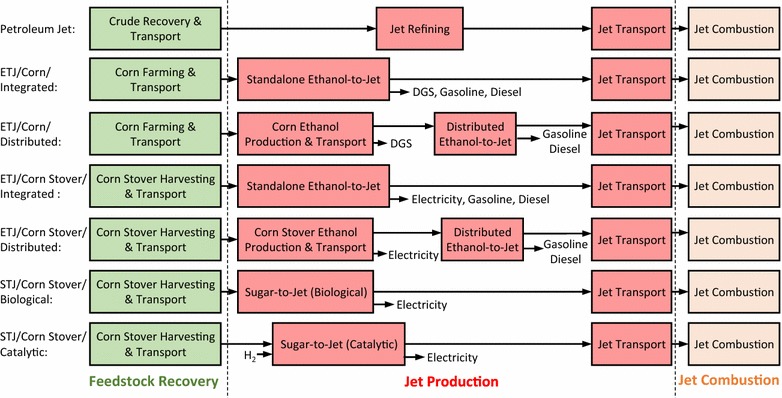
WTWa analysis system boundary (*ETJ* ethanol-to-jet, *STJ* sugar-to-jet, *DGS* distillers’ grains with solubles)

As shown in Fig. 1, the WTWa analysis system boundary in this study includes feedstock recovery (e.g., crude recovery, corn farming and harvesting, and corn stover harvesting), feedstock transport, fuel production (e.g., petroleum refining to jet, ethanol production, ETJ production, and STJ production), fuel transportation and distribution, and aircraft fuel combustion. The fuel combustion stage is also referred to as the pump-to-wake (PTWa) stage, while the rest of the stages together (so-called the upstream stages) are the well-to-pump stage.

Two feedstocks were considered for the ETJ pathways: corn and corn stover. Also, for each feedstock, two options for plant designs were examined: integrated and distributed. An integrated ETJ plant takes corn or corn stover as a feedstock, while a distributed ETJ plant takes ethanol. In other words, in a distributed ETJ production, ethanol from ethanol plants is transported to a distributed ETJ plant. Thus, the GHG emissions of ETJ from a distributed plant depend on ethanol source, which, in turn, depends on feedstocks. In particular, corn ethanol can be produced in dry or wet mills. Recently, many dry mills have adopted corn oil (CO) extraction to produce an additional by-product (CO for biodiesel production) with reduced energy consumption of distillers’ grains with solubles (DGS) drying. The impact of these ethanol sources is discussed in “Impact of corn ethanol source on WTWa GHG emissions of distributed ETJ production” section.

For the STJ pathways, corn stover was assumed as a feedstock. Note that the TEA studies, from which this study derives conversion process energy use, assumed a blended cellulosic biomass feedstock consisting of multi-pass harvested corn stover, single-pass harvested corn stover, and switchgrass. The present study assumed that the processes consume the same amount of energy if a single corn stover feedstock rather than a blended feedstock is used. It is important to note that feedstock characteristics (such as chemical compositions and ash and mineral contents) could affect product yields, energy/chemical inputs, and pretreatment requirements [34]. The impacts of these parametric assumptions on WTWa results are discussed by conducting a sensitivity analysis. It needs to be noted that STJ produced via catalytic conversion consumes a large amount of hydrogen. Thus, the source of hydrogen could substantially affect the GHG emissions associated with STJ. In order to assess the impact of hydrogen source, three hydrogen sourcing options—external H_2_ from NG steam methane reforming (SMR), in situ H_2_ from reforming of a fraction of the biomass hydrolysate, and internal H_2_ via biomass gasification—were examined.

These ETJ and STJ pathways produce several co-products. In the ETJ pathway, the corn ethanol process co-produces DGS and CO, while the corn stover ethanol and the STJ processes co-produce electricity. Also, the ETJ and STJ processes co-produce a range of liquid hydrocarbon fuels, including jet. Therefore, the co-product handling method could affect the WTWa analysis results substantially [35]. Two methods are widely used to handle co-products: displacement and allocation methods. In a displacement method, all energy and emission burdens are allocated to the main product, while the energy and emissions of producing the otherwise displaced products are taken as credits for the main products. On the other hand, an allocation method allocates the energy and emission burdens of a pathway among the products by their output shares. An appropriate choice of allocation basis is important for allocation methods. Among various allocation metrics (e.g., energy, mass, and market value), energy is often used among energy products.

This study used a hybrid approach to handle various products from different processes: a displacement method was applied for electricity and DGS while an energy allocation method was used among the hydrocarbon fuels from the ETJ and STJ processes (e.g., gasoline, jet, and diesel). This study assumed that co-produced electricity would displace the US average electricity and the US average animal meal, respectively. A displacement method was selected for DGS since an allocation may not be reliable for DGS due to the difference in the types of products (meal for nutrition vs. fuel for energy). Both allocation and displacement methods are widely used in handling electricity. This study selected a displacement method as a default method because the characteristics of electricity (e.g., value, energy form) are relatively different from those of the other hydrocarbon fuels. Also, the impacts of using an energy allocation method to estimate the WTWa GHG emissions of the AJFs from corn stover (co-producing electricity) are also presented in “Impact of co-product handling method on WTWa GHG emissions of corn stover-based ETJ and STJ” section. Among the hydrocarbon fuels produced by the ETJ and STJ processes (e.g., gasoline, jet, and diesel), on the other hand, a displacement method may not be applicable because a large portion of output can be gasoline and diesel, which could result in distorted results when jet is considered as a co-product. Thus, an energy allocation was selected. Finally, CO was handled by a process-based method, where all energy and emission burdens during ethanol production except for those associated with CO recovery were allocated to ethanol [36].

The functional unit is an important factor in LCA. This study presents the results in two functional units: an energy functional unit (MJ of jet fuel) and a resource function unit (ton of corn stover). The energy functional unit is appropriate to compare compatible fuels from different sources and to show the impacts of displacing a conventional fuel with alternative fuels (ETJ and STJ vs. conventional jet). On the other hand, the resource functional unit compares different production pathways from the same source, which can address the resource utilization issue.

### Corn farming, corn stover collection, and ethanol production

Feedstocks for ETJ and STJ in this study include corn and corn stover as well as ethanol from these feedstocks, whose key WTWa parameters are summarized in Table 2. The key parameters are based mainly on the analysis by Wang et al. [37] and subsequent updates on fertilizer applications from the latest survey of corn farming by the US Department of Agriculture (USDA) [38], corn ethanol production process updates by Mueller and Kwik [39], and the implementation of CO extraction in dry milling corn ethanol plants examined by Wang et al. [36]. In the corn stover collection stage, we assume that the supplemental fertilizer is applied to replace the nutrients in the harvested corn stover. The water consumption for corn farming includes only anthropogenic water consumption, which is the irrigation withdrawal minus the irrigation runoff [40]. We assume that corn stover does not consume water since irrigation is mainly for corn farming not for corn stover harvesting. Also, the water consumption for the ethanol production is the net of water withdrawal minus treated water returned to the same withdrawal source.

**Table 2:** Key WTWa parameters for corn and corn stover ethanol pathways

Parameter (unit)	Corn	Corn stover
Corn farming/corn stover collection (per dry ton of corn or corn stover, except as noted)		
Direct energy use (MJ)	466^a^	224^b^
N fertilizer application (kg)	19.4^b^	7.72^b^
P fertilizer application (kg)	6.70^b^	2.20^a^
K fertilizer application (kg)	6.95^b^	13.2^a^
Limestone application (kg)	52.8^a^	
N_2_O conversion rate of N fertilizer (%)	1.525^a^	
Water consumption (kL)	25.4^c^	0^c^

Parameter (unit)^c^	Dry mill w/o CO extraction	Dry mill w/CO extraction	Wet mill	Corn stover
Corn/corn stover ethanol production				
Ethanol yield (L/dry ton of corn or corn stover)	486^b^	471^b^	496^b^	375^a^
Ethanol plant fossil energy use (MJ/L of ethanol)	7.49^a^	7.36^b^	13.2^a^	
Water consumption (L/L of ethanol)	2.7^c^	2.7^c^	3.92^c^	5.35^c^
DGS yield (dry kg/L of ethanol)	0.675^a^	0.646^b^		
Corn gluten meal yield (dry kg/L of ethanol)			0.147^a^	
Corn gluten feed yield (dry kg/L of ethanol)			0.632^a^	
CO yield (dry kg/L of ethanol)		0.023^b^	0.117^a^	
Electricity yield (kWh/dry ton of corn stover)				226^a^
Enzyme use (g/dry kg of corn or corn stover)	1.04^a^	1.04^b^	1.04^a^	15.5^a^
Yeast use (g/dry kg of corn or corn stover)	0.36^a^	0.36^b^	0.36^a^	2.49^a^
Corn ethanol shares (%)	18^b^	73^b^	9^b^	

^a^Based on Wang et al. [37]

^b^Based on Wang et al. [36]

^c^Based on Lampert et al. [40]

A key issue in biofuel LCA is the impact of LUC. Especially, the LUC-related GHG emissions have been extensively discussed and evaluated since they were first estimated by Searchinger et al. [41]. While the improvements in LUC modeling and assumptions have generally lowered the estimates on LUC-related GHG emissions from the results by Searchinger et al. [41], notable variation exists among recent studies depending on LUC models, scenarios, and assumptions (see Additional file 1: Figure A1). Since the LUC-related GHG emissions were not the main focus of this study, this study employed the LUC GHG emissions by Qin et al. [42, 43], which documented detailed modeling of LUC and associated GHG emissions of ethanol pathways, including tillage (i.e., conventional, reduced, and no tillage), corn stover removal (i.e., at 0, 30, and 60% removal rates), and organic matter input techniques (i.e., cover crop and manure application). As a baseline assumption, this study used 8 and −0.7 g CO_2_/MJ ethanol for the LUC impacts of corn and corn stover ethanol, respectively, assuming conventional tillage, 30% corn stover removal, and no organic matter input techniques. Acknowledging the variations in the LUC impact, this study also conducted a sensitivity analysis using the ranges of the LUC emissions estimated by Qin et al. [42]: 5 to 17 and −1.4 to −0.6 g CO_2_e/MJ for corn and corn stover ethanol, respectively. Note that these ranges do not represent parametric uncertainty rather sensitivity around different scenarios (e.g., tillage types, soil depth, and soil carbon database).

### ETJ production

The first step in producing “drop-in” bio-jet fuel from ethanol is to remove the oxygen from the ethanol molecules via a catalytic dehydration process, producing ethylene. Then, ethylene is turned into linear or non-linear (branched) α-olefins through the catalytic oligomerization process. Depending on the oligomerization reaction chemistry (operating conditions and catalysts), the α-olefin produces a hydrocarbon distribution of C_4_ to C_32_. Because olefins are only allowed in limited quantities in jet fuel, the last upgrading step is to hydrogenate the α-olefins to produce paraffins. Then, a hydroisomerization step can be applied optionally to convert normal paraffin to their isomers. Although the C_9_–C_16_ alkanes distilled from the hydrogenated paraffins are suitable for jet fuels, key specifications for fuel properties should be used to verify whether the produced jet blendstock meets ASTM or other standards. These three upgrading steps (alcohol dehydration, olefin oligomerization, and α-olefin hydrogenation) are well-known industrial technologies and have been used for years at commercial scales. However, these processes have not been integrated into existing biorefineries to produce jet fuel. This integration may include either retrofitting existing dry mill plants to convert alcohols to jet fuel on site, or building dedicated plants that produce jet blendstocks via alcohol intermediates.

Table 3 summarizes the parametric assumptions for ETJ production processes, which are based on the TEA conducted by Wang et al. [30] on upgrading the biomass-derived ETJ blendstocks. The TEA was conducted for integrated plants at the scale of 2,000 dry metric tons of feedstock (corn or corn stover) per day. While the biochemical cellulosic ethanol model of Humbird et al. [44] was used for the front-end process of the corn stover ETJ model, the USDA corn grain dry mill model was used as the front-end process of the dry-mill-to-alcohol process [45]. Since the USDA’s corn dry mill model was developed, corn ethanol production processes have undergone technological advancements, and several studies on corn ethanol production processes reflect recent corn ethanol production trends [36, 37, 46–50]. In order to use corn ethanol production parameters reflective of current technology, the back-end ETJ process for converting ethanol-to-jet fuel was modeled separately from the integrated ETJ process. The back-end process includes ethanol dehydration, oligomerization, hydrotreating, and product fractionation, whose parametric assumptions are shown in the last column of Table 3.

**Table 3:** Parametric assumptions for ETJ fuel production processes

Feedstock	ETJ—integrated		ETJ—distributed
	Corn	Corn stover	Ethanol
Jet fuel yield (MJ jet/kg feedstock)	6.78	4.71	18.1
Natural gas use (kJ/MJ jet)	439	–	–
Hydrogen use (kJ/MJ jet)	81.3	80.9	80.9
Electricity use (Wh/MJ jet)	27.3	–	9.3
Yeast use (g/MJ jet)	0.051	–	–
Enzyme and chemical use (g/MJ jet)	1.67	26.2	–
Catalyst use (g/MJ jet)	0.094	0.107	0.107
Water use (L/L jet)	8.5	13.5	1.9
Gasoline yield (kJ/MJ jet)	210	212	212
Diesel yield (kJ/MJ jet)	113	115	115
DGS yield (dry g/MJ jet)	57	–	–
Electricity yield (Wh/MJ jet)	–	32	–

### STJ production

This study is based on two TEA studies on STJ conversion processes, which addressed the biological and catalytic conversion routes [31, 32]. In the biological conversion route, biomass feedstock is first processed in an alkaline deacetylation step to solubilize and remove acetate and other non-fermentable components, and treated with dilute sulfuric acid catalyst to liberate the hemicellulose sugars and break down the biomass for enzymatic hydrolysis. Ammonia is then added to the whole pretreated slurry to raise its pH for enzymatic hydrolysis. The hydrolyzed slurry is then filtered to remove insoluble solids (namely, lignin). The solids fraction exiting the filter is combusted to produce process heat and electricity. The remaining soluble sugar stream is split into a small fraction that is sent directly to the fed-batch bioreactors to initiate conversion and a larger fraction that is concentrated in evaporators to concentrate the sugar components. The concentrated sugar slurry from the evaporators is cooled and inoculated with the generic bioconversion microorganism under aerobic reactor conditions. Once conversion is completed, most of the cellulose and xylose are converted to free fatty acids (FFAs). Then, the FFA product is recovered via decantation and centrifugation, and hydrotreated to produce hydrocarbon fuels. In the original TEA, the primary product is a diesel-range paraffinic product suitable as a diesel blendstock. For ASTM-certified jet fuel production, the diesel-range paraffinic product needs to be hydroprocessed to saturate double-bonds. Thus, the process engineering model was adjusted to produce a jet fuel blendstock. The adjusted parametric assumptions for STJ production processes via a biological route are presented in Table 4.

**Table 4:** Parametric assumptions for STJ fuel production processes

	Biological [31]	Catalytic [32]		
		External H_2_	In situ H_2_	Gasification H_2_
Jet fuel yield (MJ jet/kg corn stover)	4.42	8.39	4.85	5.60
Hydrogen use (kJ/MJ jet)	123	528	–	–
Electricity use (Wh/MJ jet)	–	–	1.81	–
Enzyme and chemical use (g/MJ jet)	15.9	8.48	14.7	9.45
Catalyst use (g/MJ jet)	–	0.0034	0.0040	0.0036
Water use (L/MJ jet)	15.9	6.1	10.2	11.9
Electricity yield (Wh/MJ jet)	22.3	12.6	–	2.8

In the catalytic conversion route, biomass feedstock is processed by pretreatment and enzymatic hydrolysis steps similar to those of the biological conversion route. The glucose and other sugars from the hydrolysate, however, are then filtered to remove insoluble solids, concentrated by evaporation, and purified by microfiltration and ion exchange prior to catalytic upgrading, which consists of four stages: hydrogenation, aqueous-phase reforming, condensation and oligomerization, and hydrotreating. In each stage, hydrogen is required to the reactors, which operate at varying process conditions and have varying catalyst composition. The goal of these successive catalytic steps is to remove oxygen or “de-functionalize” carbohydrates and other carbon components and oligomerizes them to primarily diesel-range hydrocarbons. All four stages in catalytic upgrading consume a large amount of hydrogen. Davis et al. [32] investigated three hydrogen sources: external H_2_ from NG SMR, in situ H_2_ produced by reforming a fraction of biomass hydrolysate, and internal H_2_ produced by biomass gasification. Table 4 presents the parametric assumptions for STJ fuel production processes via the catalytic conversion route with three different hydrogen sources. Note that the internal hydrogen production reduces jet fuel production significantly because a fraction of biomass or its derivatives is used for hydrogen production rather than jet fuel production. It also should be noted that this LCA study did not make any modifications to the sugar catalytic upgrading TEA model or its associated cost results in Davis et al. [32].

### Crude oil recovery and petroleum jet fuel production

The system boundary of petroleum jet fuel includes crude oil recovery and transport, and jet fuel refining, transportation, distribution, and combustion. More than half of total crude oil refined in the US refining sector in 2015 (54%) was produced domestically [1]. The foreign sources of crude include Canada (18%), Middle East (12%), Mexico (5%), Latin America (9%), and other regions (2%). Among them, Canadian crude consists of conventional crude and crude from oil sands, accounting for 10 and 8%, respectively, of the total crude supply to US refineries. Note that the oil sands’ share of the crude mix is an important WTWa analysis parameter because of the high GHG intensity of oil sands compared to conventional oil. Cai et al. [51] investigated the GHG intensities of oil sands products from four different production technologies, including the GHG emissions from land disturbance associated with oil sands recovery. Another crude source that has recently gained significant importance in the US is shale oil. The share of shale oil production as a fraction of the total crude production in the US has increased from 14% in 2010 to 48% in 2015 [52]. The present study estimated the energy intensity and GHG emissions of shale oil using the parameters for shale oil recovery reported by Brandt et al. [53] and Ghandi et al. [54] for the Bakken and Eagle Ford plays, respectively, while the conventional crude recovery parameters are based on those of Burnham et al. [55].

The present study used the energy consumption of jet fuel production estimated by Elgowainy et al. [56], who investigated 43 large US refineries (each with a refining capacity greater than 100,000 barrels per day) using a linear programing model. The 43 refineries represented 70% of the total US refining capacity and covered a wide range of crude sources/quality, product slates, and refinery complexity. The linear programing model generated the volumetric and mass flow rates as well as the utility consumptions of individual process units in the refineries, which were used to estimate the energy consumption for each process unit. The energy consumptions of individual process units, then, were allocated to intermediate products of the unit by their energy content in order to estimate the energy intensity of the intermediate products. By estimating the energy intensity of all streams and aggregating them for the streams that make various final products, the product-specific efficiency of petroleum products was estimated. Table 5 provides the process fuel use for jet fuel production estimated by Elgowainy et al. [56].

**Table 5:** Refinery process fuel use for major fuel products (kJ_process fuel_/MJ_fuel product_)

Purchased fuels				Internally produced fuels	
NG—SMR	NG—combustion	Electricity	H_2_	Fuel gas combustion	Catalytic coke combustion in fluid catalytic cracking
5.5	19	1.6	4.3	13	2.7

## Results

**Fig. 2:**
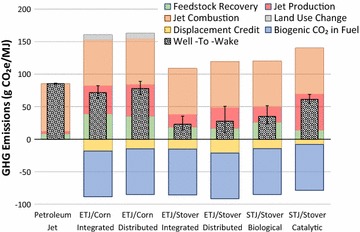
WTWa GHG emissions of ETJ and STJ compared to petroleum jet

Figure 2 presents the WTWa GHG emissions of four ETJ and two STJ pathways compared to petroleum jet. The petroleum jet generates WTWa GHG emissions of 85 g CO_2_e/MJ. The four ETJ pathways include ETJ from corn using integrated and distributed plants (denoted as ETJ/Corn/Integrated and ETJ/Corn/Distributed, respectively) and ETJ from corn stover using integrated and distributed plants (denoted as ETJ/Stover/Integrated and ETJ/Stover/Distributed, respectively). The two STJ pathways include STJ from corn stover using biological and catalytic conversion routes. For the catalytic conversion route, H_2_ is assumed to be produced externally using NG SMR. The lower and upper ends of the error bars in the figure represent the 10th and 90th percentiles of the resulting distributions from Monte Carlo simulations. The GREET model maintains 887 parameters with distribution functions defined. Among them, the distribution function definition of 27 key parameters for the ETJ and STJ pathways are provided in Additional file 1: Table A1. Note that the conversion process assumptions are point estimates without distributions. The resulting distributions are caused by the variations in the upstream stages (e.g., corn farming, corn stover harvesting, and process fuel, chemical, and enzyme production).

The WTWa emissions of each pathway are the sum of the GHG emissions from feedstock recovery, jet production, and jet combustion, and the credits from conventional product displacement and biogenic CO_2_ in fuel as well as LUC emissions. Figure 1 illustrates what the feedstock recovery, the jet production, and the jet combustion in Fig. 2 include. As explained in “WTWa analysis system boundary and methods” section, the displacement credits are the avoided GHG emissions by displacing the conventional products (animal feeds and US average electricity) with the co-products from the pathways (DGS and electricity). The biogenic CO_2_ in fuel denotes the amount of CO_2_ absorbed during biomass growth that is ended up in fuel and combusted. Since we assumed that all carbon in fuel is derived from biomass and carbon in biomass is carbon neutral, the size of the biogenic CO_2_ in fuel is almost identical to that of jet combustion, which almost cancel out each other. It should be noted that a carbon neutrality assumption for biomass with short carbon cycles (e.g., annual crops) is generally agreed while that with long carbon cycles (e.g., woody biomass) is debatable.

The WTWa GHG emissions of corn-based ETJ are estimated at 72 and 78 g CO_2_e/MJ for integrated and distributed plants, respectively, while the GHG emissions of corn stover-based ETJ are 23 and 28 g CO_2_e/MJ for integrated and distributed plants, respectively. The large GHG emissions of corn-based ETJ are caused by the high GHG intensity of corn farming and corn ethanol production as well as LUC. Corn farming consumes a large amount of fertilizer, especially nitrogen fertilizer. Nitrogen fertilizer production is highly energy- and GHG-intensive and generates a significant amount of N_2_O emission once it is applied on farm fields. Corn ethanol production is also quite energy- and GHG-intensive, consuming a significant amount of process fuels (mainly NG). Note that there are some options to reduce GHG emissions of corn ethanol, which can be applied to the corn-based ETJ pathways: (1) replacement of NG with biogas in ethanol plants and (2) integrated corn and corn stover ethanol production analyzed in Canter at el. [50]. On the other hand, corn stover harvesting requires only a small amount of fertilizers for supplementing nutrient losses from stover removal. Also, cellulosic ethanol production generates energy (heat and electricity) from lignin combustion beyond process requirements; excess electricity is exported to the grid. Feedstocks themselves vary significantly in their GHG intensities. Therefore, the feedstock needs to be clearly defined when the GHG emissions of ETJ are calculated. The conversion process used in the corn stover-based ETJ is similar to the low case of the switchgrass advanced fermentation pathway in Staples et al. [27], which showed 11.7 g CO_2_e/MJ of GHG intensity. A main driver of the lower GHG emissions estimated in Staples et al. [27] than estimates in this study is the feedstock and process fuel consumptions: Staples et al. [27] assumed about 70% lower feedstock and process fuel consumptions for the conversion process than this study.

The integrated cases of ETJ generate about 5–6 g CO_2_e/MJ lower GHG emissions than the distributed cases of ETJ because of less stringent ethanol feedstock quality and heat integration. The distributed plant is assumed to take market ethanol with moisture content less than 1% [57]. On the other hand, the ethanol feedstock in the integrated plant can contain 7.5% water, which can reduce energy consumed in distillation in ethanol production. Also, the integrated plant allows better heat integration between the ethanol and ETJ plants. Note that the removal of ethanol transport in the integrated production did not affect the GHG emissions of ETJ greatly, since its impact was offset by the longer transportation and distribution distance of ETJ from the ETJ plant to consumption.

The WTWa GHG emissions of corn stover-based STJ produced via biological and catalytic conversion are estimated at 35 and 61 g CO_2_e/MJ, respectively. The large WTWa GHG emissions of STJ via catalytic conversion result from consumption of a large amount of H_2_. “Impact of H_2_ source on WTWa GHG emissions of STJ via catalytic conversion” section discusses the impact of H_2_ source on the WTWa GHG emissions of STJ via catalytic conversion. The conversion processes used in the corn stover-based STJ via biological and catalytic conversion are similar to the base case of the switchgrass advanced fermentation pathway in Staples et al. [27] and the STJ pathway in Budsberg et al. [28]. The GHG emissions of the two pathways in these previous studies are 37.4 and 66 g CO_2_e/MJ of GHG intensity, respectively, similar to this study’s estimates.

**Fig. 3:**
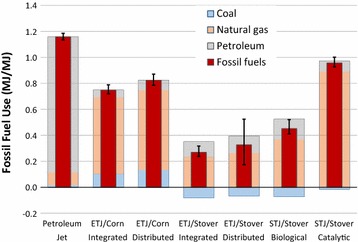
WTWa fossil fuel use of ETJ and STJ compared to petroleum jet

As presented in Fig. 3, the WTWa fossil fuel use, the sum of coal, NG, and petroleum, shows a similar trend to the WTWa GHG emissions shown in Fig. 2. A similar trend results from the fact that the majority of GHG emissions is CO_2_ from combustion of fossil fuels. However, the trend is not completely linear because of other emissions (most notably, N_2_O emissions from N fertilizers and biomass). Compared to the petroleum jet (1.16 MJ/MJ), the fossil fuel use of corn-based ETJ is estimated at 0.75 and 0.82 MJ/MJ for integrated and distributed plants, respectively, while the fossil fuel use of corn stover-based ETJ is 0.27 and 0.33 MJ/MJ for integrated and distributed plants, respectively. Also, the fossil fuel use of corn stover-based STJ produced via biological and catalytic conversion are estimated at 0.45 and 0.96 MJ/MJ, respectively. NG consumption accounts for the largest share of the fossil fuel use of the ETJ and STJ pathways. Especially, corn ethanol production for corn-based ETJ and H_2_ consumption for STJ via catalytic conversion account for the largest NG consumption. The negative coal use for the corn stover-based ETJ and STJ pathways results from the displacement of the US average generation mix, 41% of which is from coal.

**Fig. 4:**
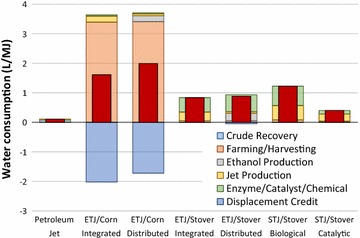
WTWa water consumption of ETJ and STJ compared to petroleum jet

Figure 4 provides the WTWa water consumption of ETJ and STJ compared to petroleum jet. For the corn-based ETJ pathways, the irrigation for corn farming is the major water consumption, accounting for approximately 3.4 L/MJ. Note that the corn-based ETJ pathways also have large water credits due to animal feed displacement. Corn stover-based ETJ from integrated and distributed plants consumes 0.83 and 0.88 L of water per MJ, respectively, while STJ via biological conversion consumes 1.2 and 0.40 L of water per MJ, respectively. For the corn stover-based ETJ and STJ pathways, water is consumed largely for enzyme production and jet fuel production.

## Discussion

### Impact of corn ethanol source on WTWa GHG emissions of distributed ETJ production

**Fig. 5:**
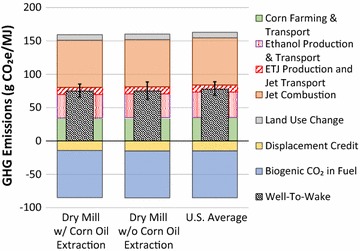
WTWa GHG emissions of ETJ with distributed production using ethanol from various sources

Currently, 208 ethanol plants in the US receive corn as a feedstock [33]. While each ethanol plant is unique, they can be categorized into three groups: dry mills with CO extraction, dry mills without CO extraction, and wet mills. Ethanol from dry mills with CO extraction, dry mills without CO extraction, and wet mills accounts for 71, 18, and 11% of US ethanol production, respectively [36]. Figure 5 presents the WTWa GHG emissions of ETJ with distributed plants using ethanol from dry mills with and without CO extraction as compared to ETJ using the US average ethanol. ETJ using ethanol from dry mills with and without CO extraction generates 75 and 75 g CO_2_e of GHG emission per MJ of ETJ, respectively. Because of the small amount of CO relative to ethanol, the impact of CO extraction on the ETJ’s GHG emissions is minimal with a process-based approach to handle the co-products (0.2 g CO_2_e/MJ). Note that the impact of CO extraction would be also small with an allocation method due to the small amount of CO as discussed in Wang et al. [36]. Note that ETJ using ethanol from wet mills is not presented because the share of energy- and GHG-intensive wet milling ethanol plants is small.

### Impact of H_2_ source on WTWa GHG emissions of STJ via catalytic conversion

**Fig. 6:**
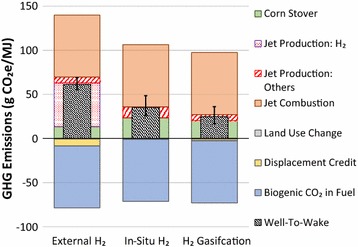
WTWa GHG emissions of STJ via catalytic conversion using H_2_ from three different sources

Because of the large quantity of H_2_ consumption in STJ production via catalytic conversion, the H_2_ source affects the WTWa GHG emissions of STJ significantly. Thus, this study examined the impact of three different H_2_ sources on the WTWa GHG emissions based on the assumptions shown in Table 4 [32]: external H_2_ from NG SMR, in situ H_2_ from biomass, and H_2_ from biomass gasification. As shown in Fig. 6, the WTWa GHG emissions of STJ via catalytic conversion are estimated at 61, 35, and 25 g CO_2_e/MJ with external H_2_ from NG SMR, in situ H_2_ from biomass, and H_2_ from biomass gasification, respectively. H_2_ used in the external H_2_ case accounts for more than 80% of the WTWa GHG emissions (49 g CO_2_e/MJ). Because a significant source of GHG emissions is removed using biomass as a H_2_ source, the in situ H_2_ and biomass gasification cases can show significantly reduced GHG emissions. The use of biomass for H_2_ production, however, lowers the jet fuel yields from 251 L/ton corn stover to 145 L/ton in the in situ H_2_ case and 157 L/ton in the biomass gasification case, which could adversely impact the economics of the STJ plants. Especially, the low NG price due to expansion of shale gas production in the US makes it not attractive to justify the production of H_2_ from biomass over NG SMR. Thus, the trade-off between the GHG emissions and economic feasibility depending on the H_2_ source needs to be examined.

### Impact of co-product handling method on WTWa GHG emissions of corn stover-based ETJ and STJ

**Fig. 7:**
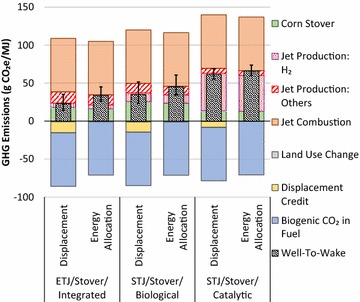
WTWa GHG emissions of corn stover-based ETJ and STJ using displacement and energy allocation methods

As mentioned earlier, the co-product handling method is an important factor in biofuel LCA because biofuel production is associated with various co-products [35]. Thus, this study examined the impact of co-product handling method on the WTWa GHG emissions of corn stover-based ETJ and STJ (where electricity is the co-product), which is presented in Fig. 7. The WTWa GHG emissions of corn stover-based ETJ and STJ estimated with the displacement method are generally lower than those estimated with the energy allocation method, by 11, 10, and 5 g CO_2_e/MJ for ETJ and STJs via biological and catalytic conversions, respectively. GHG emissions are lower when the displacement method is used, because electricity displacement credits that ethanol receives exceed the GHG emissions allocated to the electricity when the energy allocation method is used. It should be noted that the US average electricity, whose GHG intensity is estimated at 613 g CO_2_e/kWh, is assumed to be displaced. If a different generation mix for electricity is assumed, the displacement credit would be changed, resulting in different WTWa GHG emissions results. For example, if electricity produced in the Midwest Reliability Organization region (covering all of Minnesota, North Dakota, and Nebraska; portions of Montana, South Dakota, Iowa, and Wisconsin; and the Upper Peninsula of Michigan), which has a GHG intensity of 714 g CO_2_e/kWh, were displaced, WTWa GHG emissions of corn stover-based ETJ and STJ estimated with the displacement method would increase by 2.4, 2.3, and 1.3 g CO_2_e/MJ, respectively.

As mentioned in “WTWa analysis system boundary and methods” section, both displacement and energy allocation methods are widely used to handle electricity co-products. As evidenced by the overlaps of p10–p90 ranges in these corn stover-based ETJ and STJ pathways between the methods, both methods provide acceptable estimates on WTWa GHG emissions. In these pathways, the WTW GHG emissions estimated by a displacement method are reliable because the main product (hydrocarbon fuels) dominates the product slate and a conventional product to be displaced can be defined clearly. In case of jet production from a specific plant, the estimates can be further refined by using the regional electricity that is actually displaced with the co-produced electricity.

### Sensitivity analysis on key parameters of the ETJ and STJ pathways

In addition to the error bars in Figs. 2, 3 and 5, 6, 7 presenting the aggregated impacts of the variations and uncertainties associated with the pathways using the GREET stochastic modeling feature, this study conducts a sensitivity analysis to show the impacts of individual parameters on the WTWa results of these pathways. For the sensitivity analysis, the p10 and p90 values of key parameters in corn farming, corn ethanol production, corn stover collection, and corn stover ethanol production shown in Additional file 1: Table A1 were used. As mentioned in “Corn farming, corn stover collection, and ethanol production” section, LUC-related GHG emissions ranges estimated by Qin et al. [42] were also examined. Due to lack of reliable range estimates, this study perturbed the other key parameters by ±10% to conduct the sensitivity analysis. The other key parameters include irrigation in corn farming, water use in corn and corn stover ethanol production, jet fuel, and electricity yield in jet production, and usage intensities of NG, H_2_, electricity, yeast, enzyme/chemical, catalyst, and water in jet production.

Note that changing one parameter could affect other parameters. For example, increasing jet yield in jet production could require additional energy and H_2_ uses and lowers co-product yields, which require a process engineering analysis or TEA. Since this sensitivity analysis is intended to present the individual impact of each parameter rather than assessing the sensitivity of a different scenario, this sensitivity analysis treats the perturbation of each parameter independently.

Additional file 1: Figure A2 provides the sensitivity analysis results of GHG emissions on key parameters of the ETJ and STJ pathways. The values in the parenthesis for each parameter denote the values resulting in the low, base and high GHG emissions results. For all of the ETJ and STJ pathways investigated in this study, the most influential parameter is N_2_O conversion rate of N fertilizers due to the high global warming potential of N_2_O. For the corn-based ETJ pathways, the LUC GHG emissions are also considerably important as these emissions are highly uncertain. Other important parameters to the WTWa GHG emissions include the N fertilizer application rate, and the jet fuel yields in jet production.

For fossil fuel use (presented in Additional file 1: Figure A3), the jet fuel yields in jet production and the N fertilizer application rate are critical factors in general. Three exceptions include the corn-based ETJ pathways, the corn stover-based ETJ pathway with distributed plants, and the STJ pathway via catalytic conversion with external H_2_, which are sensitive to the energy use in ethanol or jet production, the electricity yield in ethanol production, and the H_2_ consumption in jet production, respectively.

As shown in Additional file 1: Figure A4, the water consumptions of the corn-based ETJ pathways depends largely on the irrigation in corn farming. The jet fuel yield is also important for the corn-based ETJ pathways because of the high water intensity of corn. Moreover, DGS yield in jet production in integrated plants can affect the WTWa water consumption since DGS displaces water-intensive animal feeds. On the other hand, the WTWa water consumptions of the corn stover-based ETJ and STJ pathways do not vary significantly.

### WTWa GHG emissions of ETJ and STJ per ton of corn stover

The WTWa GHG emissions results above are presented on a per-MJ basis, which is informative when comparing similar fuels from different sources (e.g., petroleum jet, NG-based FTJ, HEFA, and other bio-aviation fuels). These per-MJ results, however, may not address resource utilization issues, such as which pathway can bring about the greatest reduction in GHG emissions and displace the largest amount of petroleum using one ton of corn stover. As shown in “Impact of H_2_ source on WTWa GHG emissions of STJ via catalytic conversion” section, the two STJ cases involving catalytic conversion with internal H_2_ from biomass have lower GHG emissions but yield a smaller amount of liquid fuel than STJ produced via catalytic conversion with external H_2_ from NG SMR. Because of the low liquid fuel yields, the pathway with lower GHG emissions on a per-MJ basis could have higher GHG emissions on a per-ton-of-biomass basis.

**Fig. 8:**
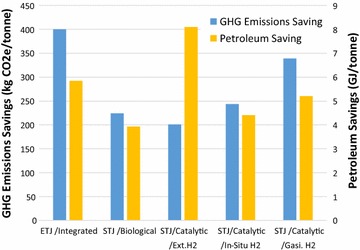
WTWa GHG emissions savings and petroleum savings of corn stover-based ETJ and STJ

To address this resource utilization issue, Fig. 8 presents the WTWa GHG emissions and petroleum savings of corn stover-based ETJ and STJ in kg CO_2_e and GJ per dry ton corn stover, respectively. WTWa GHG emissions savings of ETJ, STJ via biological conversion, and STJ via catalytic conversion using external H_2_, in situ H_2_, and H_2_ from biomass gasification are 320, 223, 200, 244, and 339 kg CO_2_e/dry ton corn stover, respectively, while the WTWa petroleum savings are 5.8, 3.9, 8.1, 4.4, and 5.2 GJ/ton, respectively. The largest GHG emissions and petroleum savings result from the displaced hydrocarbon fuels (jet, gasoline, and diesel). The savings from displaced hydrocarbon fuels include the avoided energy use and emissions associated with both production and use of the displaced hydrocarbon fuels. Except for STJ produced via catalytic conversion with external H_2_, the GHG emissions and petroleum savings are directionally correlated. However, STJ produced via catalytic conversion with external H_2_ shows very large petroleum savings because of its high liquid fuel yield, but shows small GHG emissions savings because of its large H_2_ consumption. Thus, a trade-off between GHG emissions and petroleum savings exists for STJ produced via catalytic conversion with different H_2_ sources.

## Conclusions

This study examined various emerging AJF pathways (e.g., ETJ and STJs produced via biological and catalytic conversions) and showed that the WTWa GHG emissions reductions achieved with corn stover-based ETJ with integrated production could be 73% relative to petroleum jet. For corn-based ETJ with integrated production, on the other hand, the GHG emissions are reduced by 16% relative to petroleum jet. Moreover, corn- and corn stover-based ETJ with integrated production could reduce the fossil fuel use by 35 and 77%, respectively. However, ETJ consumes a much larger amount of water than petroleum jet due to irrigation for corn farming and enzyme production for corn stover-based ETJ production, resulting in the water consumption at 1.6–1.9 L/MJ of corn-based ETJ and 0.83–0.88 L/MJ of corn stover-based ETJ. It should be noted that ETJ offers unique opportunities to exploit extensive existing corn ethanol plants and infrastructure, and to provide a boost to staggering ethanol demand, which is largely being used as gasoline blendstock.

This study also showed that STJ synthesized via biological conversion could reduce GHG emissions by 59% relative to petroleum jet. On the other hand, the GHG emission reduction achieved with STJ synthesized via catalytic conversion depends highly on the H_2_ source. The GHG emissions of STJ synthesized via catalytic conversion can be reduced up to 71% relative to petroleum jet with H_2_ from biomass gasification while external H_2_ from NG SMR would result in 28% WTWa GHG emissions reduction relative to petroleum jet. However, the external H_2_ case results in a much higher liquid fuel yield. Thus, there is a clear trade-off between GHG emissions and petroleum savings when the pathways are compared on a per-ton-of-corn stover basis. The fossil fuel use of STJ via biological and catalytic conversion with external H_2_ is 61 and 17% lower than that of petroleum jet, respectively, while their water consumption is estimated at 1.23 and 0.40 L/MJ, respectively.

Since the co-product handling method is a critical LCA issue, this study examined the impacts of co-product handling methods (i.e., displacement and energy allocation methods) on WTWa GHG emissions of corn stover-based ETJ and STJ, and showed that the choice of co-product handling method can change the WTWa GHG emission results by up to 11 g CO_2_e/MJ. Thus, careful consideration of the co-product handling method is warranted in examining or comparing different AJF pathways. Also, this study investigated only STJ processes that combust all lignin and co-produce electricity. Lignin, however, could be converted to chemicals including adipic acid, butadiene, butanediol, and cyclohexane to improve process economics [31]. In such cases, further examination of co-product treatment is needed.

## Additional file

**Additional file 1**. Additional file 1 includes a summary of key LUC-related emissions results from previous studies (Figure A1), the distribution function definition of 27 key parameters for the ETJ and STJ pathways (Table A1), and the sensitivity analysis results of key parameters for the ETJ and STJ pathways (Figures A2 to A4).

## Abbreviations

AJF: alternative jet fuel
GHG: greenhouse gas
WTWa: well-to-wake
ETJ: ethanol-to-jet
STJ: sugar-to-jet
HEFA: hydroprocessed ester and fatty acids
FTJ: Fischer–Tropsch jet
ATJ: alcohol-to-jet
HRJ: hydroprocessed renewable jet
NG: natural gas
ASTM: American Society for Testing and Materials
LUC: land use change
LCA: life-cycle analysis
TEA: techno-economic analysis
DGS: distillers’ grains with solubles
PTWa: pump-to-wake
CO: corn oil
SMR: steam methane reforming
USDA: US Department of Agriculture
