# Supplementary material for: Well-to-wake analysis of ethanol-to-jet and sugar-to-jet pathways
Source: Biotechnol Biofuels. 2017 Jan 24;10:21. doi: 10.1186/s13068-017-0698-z (PMC5260116; doi:10.1186/s13068-017-0698-z)
Supplement: Supplementary file 1 — Additional file 1. Additional file 1 includes a summary of key LUC-related emissions results from previous studies (Figure A1), the distribution function definition of 27 key parameters for the ETJ and STJ pathways (Table A1), and the sensitivity analysis results of key parameters for the ETJ and STJ pathways (Figures A2 to A4). [file 13068_2017_698_MOESM1_ESM.pdf]

Well-To-Wake Analysis of Ethanol-To-Jet and Sugar-To-Jet Pathways

Jeongwoo Han,[a] Ling Tao,[b] Michael Wang[a]

[a] Systems Assessment Group, Energy Systems Division, Argonne National Laboratory, 9700 S. Cass Avenue, Argonne, IL 60439

[b] National Renewable Energy Laboratory, 15013 Denver West Parkway, Golden, CO 80401

Additional file 1

This Additional file 1 includes a summary of key LUC-related emissions results from previous studies, the distribution function definition of 27 key parameters for the ETJ and STJ pathways, and the sensitivity analysis results of key parameters for the ETJ and STJ pathways.

Summary of key LUC-related emissions results of corn ethanol from previous studies

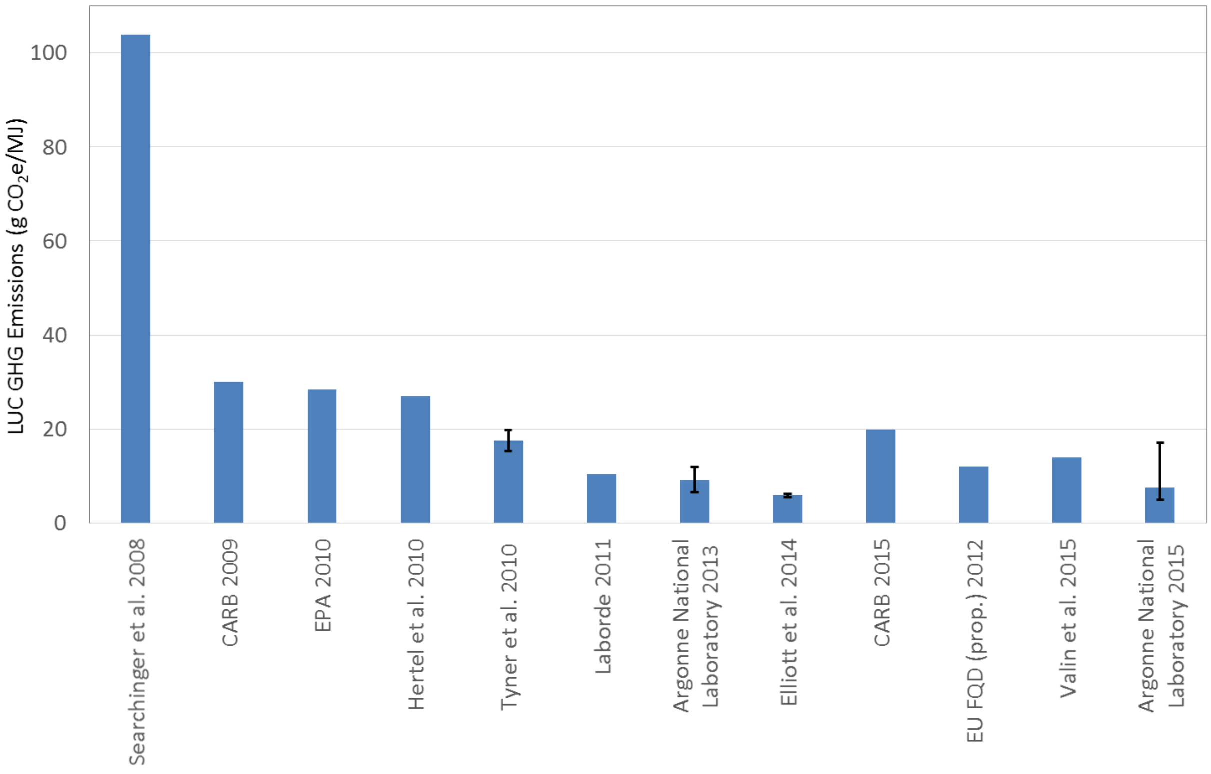

Figure A1 Estimates for LUC contribution to GHG emissions from corn ethanol production (g CO_2e/MJ) [1–12]

Distribution function definition of 27 key parameters for the ETJ and STJ pathways

Table A1 Distribution function definition of 27 key parameters for the ETJ and STJ pathways

	Mean	P10	P90	Distribution function type
**Corn farming**				
Direct energy use (MJ)[a]	466	382	585	Weibull
N fertilizer application (kg)[b]	19.4	14.8	24.1	Normal
P fertilizer application (kg)[b]	6.70	3.53	10.61	Lognormal
K fertilizer application (kg)[b]	6.95	1.66	13.59	Weibull
Limestone application (kg)[b]	52.8	47.5	58.1	Normal
N_2O conversion rate of N fertilizer (%)[b]	1.525	0.413	2.956	Weibull
**Corn ethanol production: Dry mill w/o CO extraction**				
Ethanol yield (L/dry tonne of corn)[b]	486	471	496	Triangular
Ethanol plant fossil energy use (MJ/L of ethanol)[a]	7.49	5.55	9.42	Normal
DGS yield (dry kg/L of ethanol)[a]	0.675	0.609	0.743	Triangular
Enzyme use (g/dry kg of corn)[a]	1.04	0.936	1.15	Normal
Yeast use (g/dry kg of corn)[a]	0.36	0.32	0.40	Normal
**Corn ethanol production: Dry mill w/ CO extraction**				
Ethanol yield (L/dry tonne of corn)[b]	490	475	500	Triangular
Ethanol plant fossil energy use (MJ/L of ethanol)[a]	7.36	5.46	9.26	Normal
Enzyme use (g/dry kg of corn)[a]	1.04	0.936	1.15	Normal
Yeast use (g/dry kg of corn)[a]	0.36	0.32	0.40	Normal
**Corn ethanol production: Wet mill**				
Ethanol yield (L/dry tonne of corn)[b]	454	439	463	Triangular
Ethanol plant fossil energy use (MJ/L of ethanol)[a]	13.2	11.5	14.9	Normal
Enzyme use (g/dry kg of corn)[a]	1.04	0.936	1.15	Normal
Yeast use (g/dry kg of corn)[a]	0.36	0.32	0.40	Normal
**Corn stover collection**				
Direct energy use (MJ)[b]	224	201	246	Normal
N fertilizer application (kg)[b]	7.72	5.91	9.52	Normal
P fertilizer application (kg)[a]	2.20	1.10	3.31	Normal
K fertilizer application (kg)[a]	13.2	7.5	19.0	Normal
**Corn stover ethanol production**				
Ethanol yield (L/dry tonne of corn stover)[a]	375	328	423	Normal
Electricity yield (kWh/dry tonne of corn stover)[a]	226	162	290	Triangular
Enzyme use (g/dry kg of corn stover)[a]	15.5	9.6	23	Triangular
Yeast use (g/dry kg of corn stover)[a]	2.49	2.24	2.74	Normal

[a] Based on Wang et al. [13].
[b] Based on Wang et al. [14].

Sensitivity analysis results on key parameters for the ETJ and STJ pathways

GHG Emissions in g CO_2e/MJ

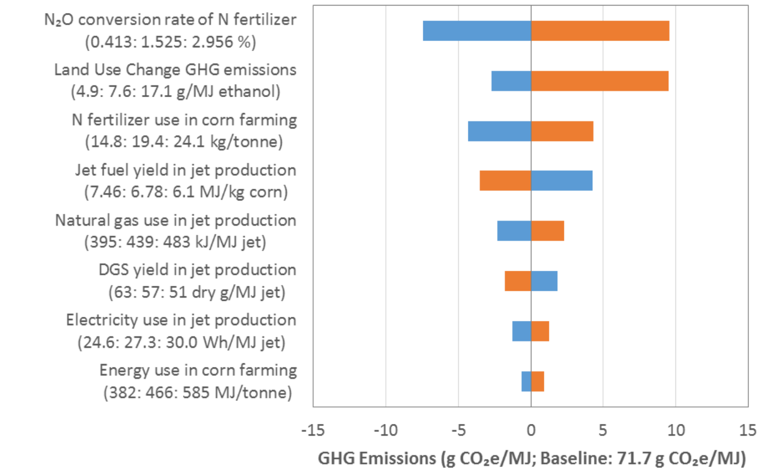

(a) Corn-based ETJ using integrated plants

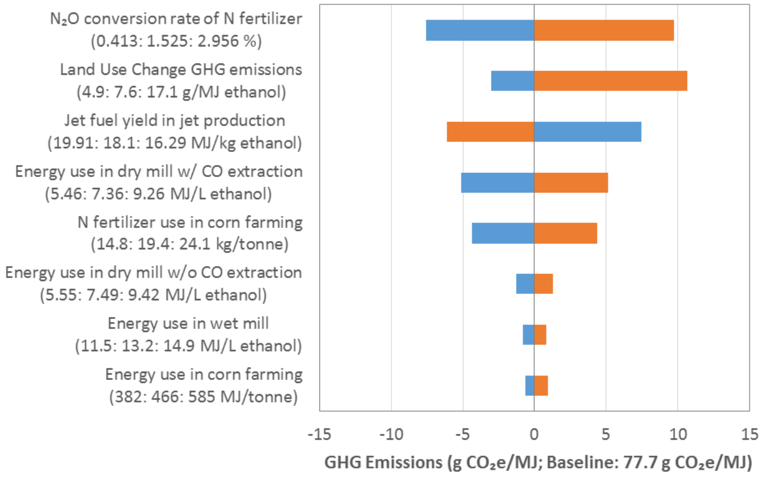

(b) Corn-based ETJ using distributed plants

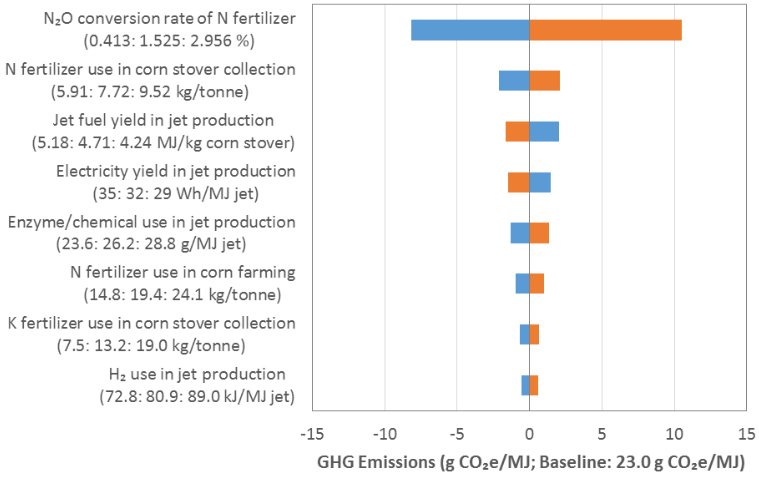

(c) Corn stover-based ETJ using integrated plants

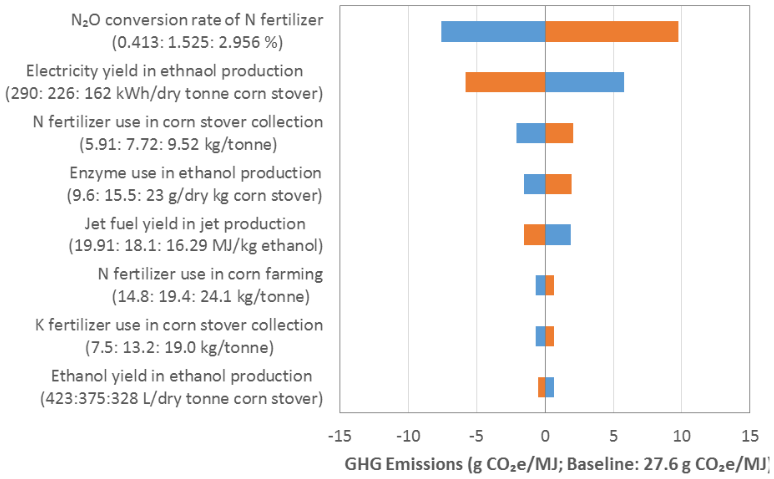

(d) Corn stover-based ETJ using distributed plants

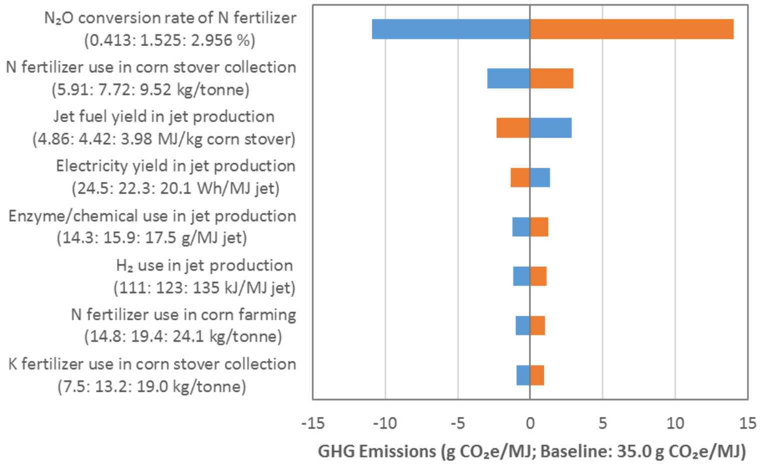

(e) Corn stover-based STJ via biological conversion

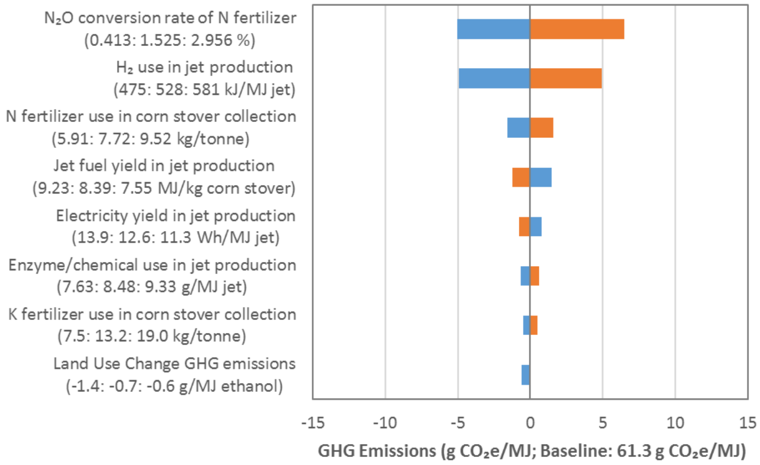

(f) Corn stover-based STJ via catalyst conversion with external H_2

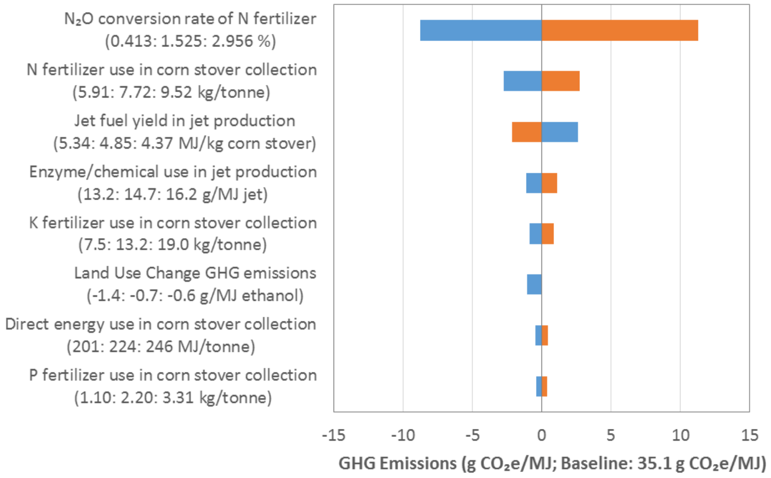

(g) Corn stover-based STJ via catalyst conversion with In-Situ H_2

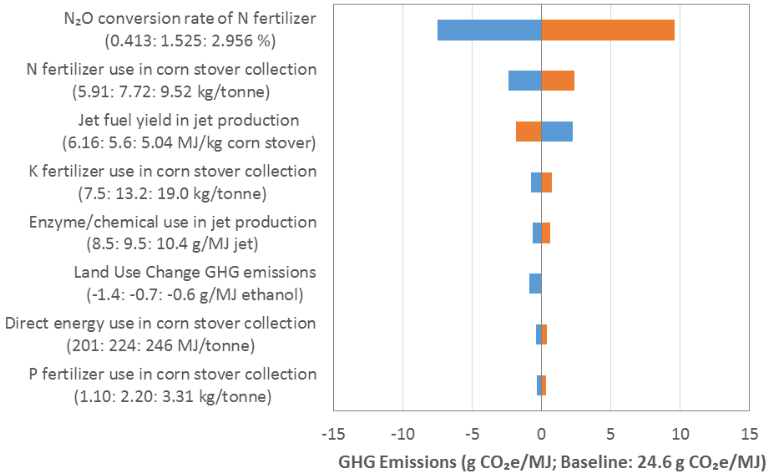

(h) Corn stover-based STJ via catalyst conversion with gasification H_2

Figure A2 Sensitivity analysis of GHG emissions on key parameters of the ETJ and STJ pathways in g CO_2e/MJ

Fossil Fuel Use in MJ/MJ

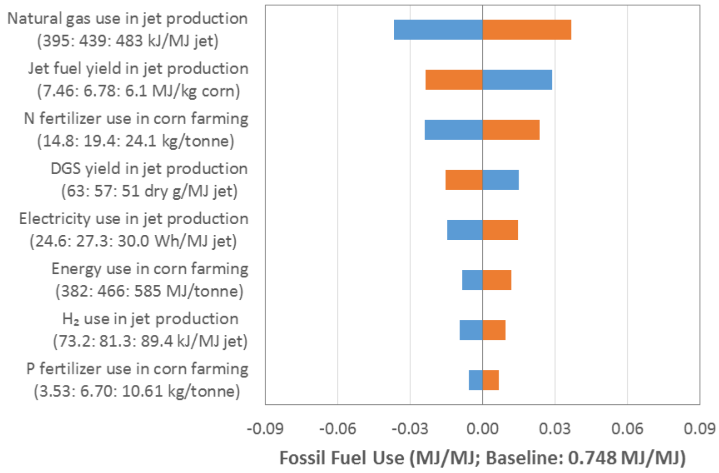

(a) Corn-based ETJ using integrated plants

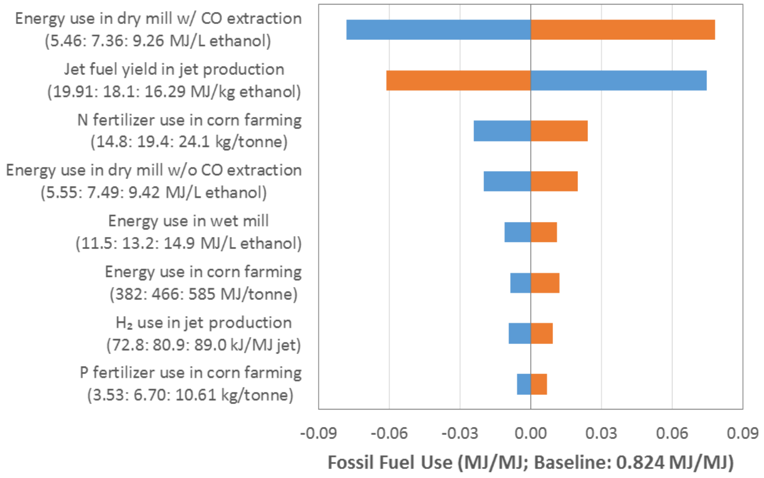

(b) Corn-based ETJ using distributed plants

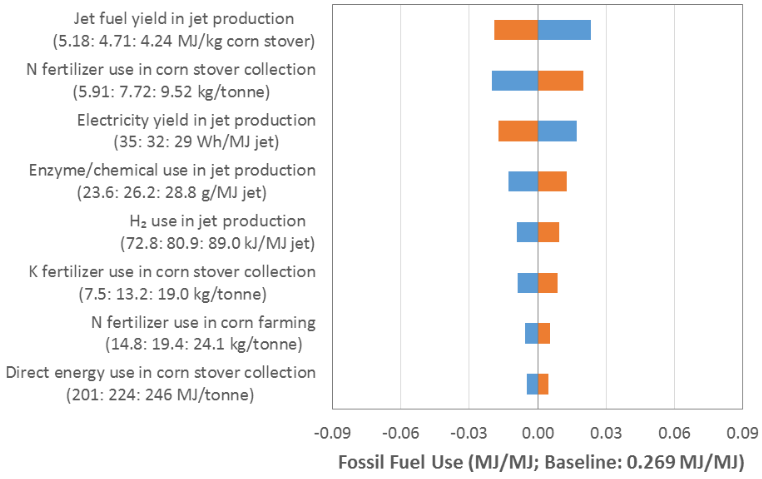

(c) Corn stover-based ETJ using integrated plants

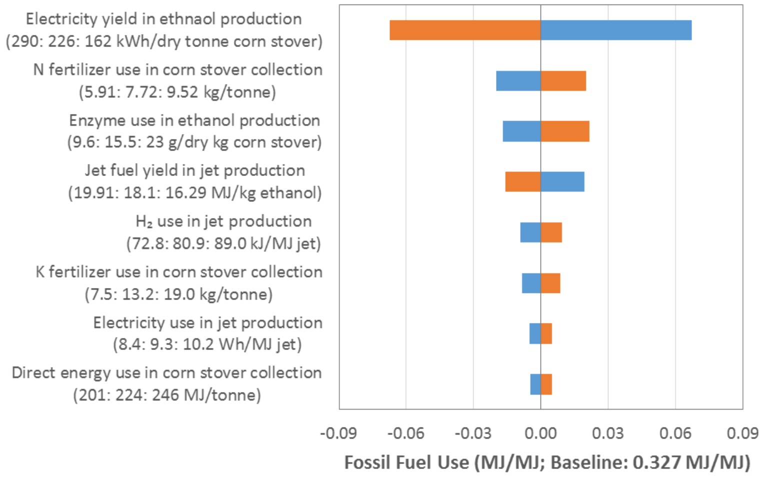

(d) Corn stover-based ETJ using distributed plants

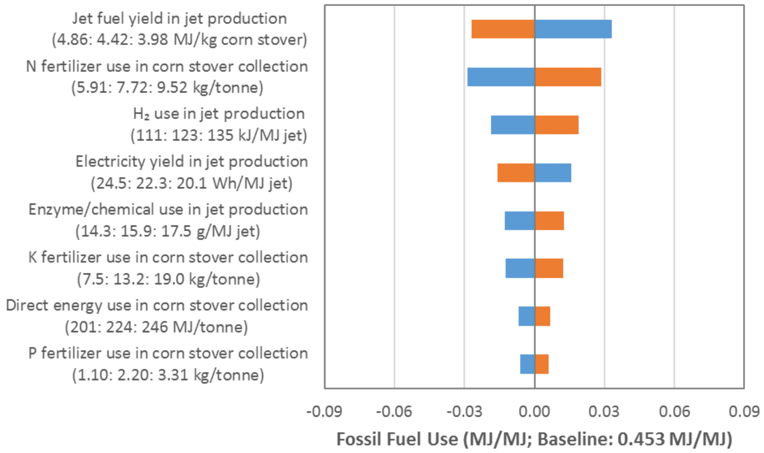

(e) Corn stover-based STJ via biological conversion

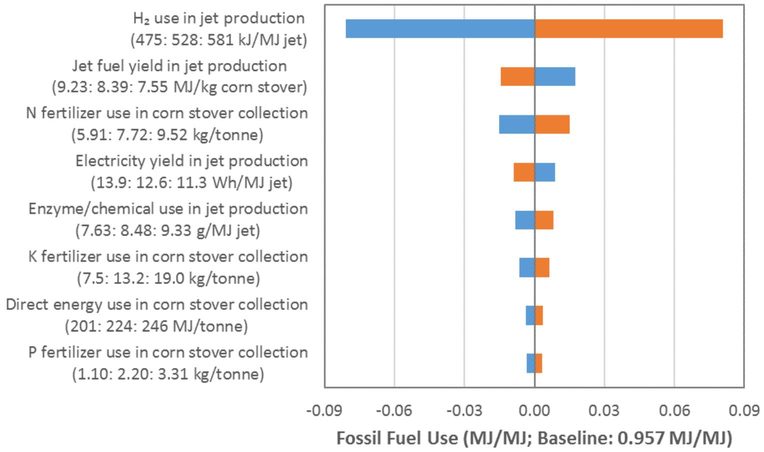

(f) Corn stover-based STJ via catalyst conversion with external H_2

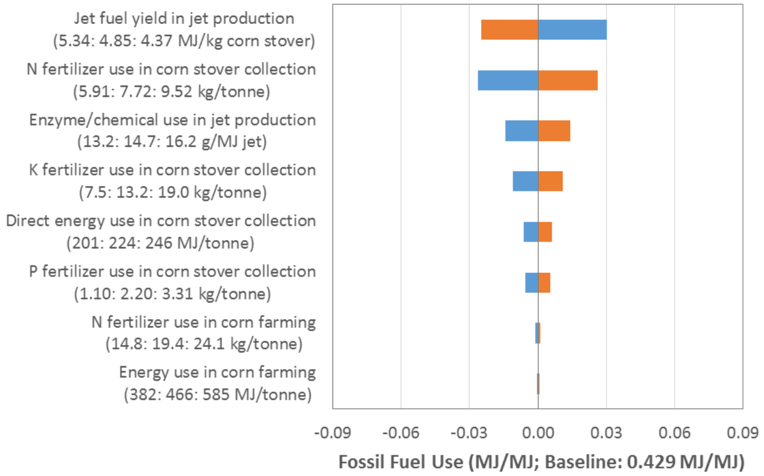

(g) Corn stover-based STJ via catalyst conversion with In-Situ H_2

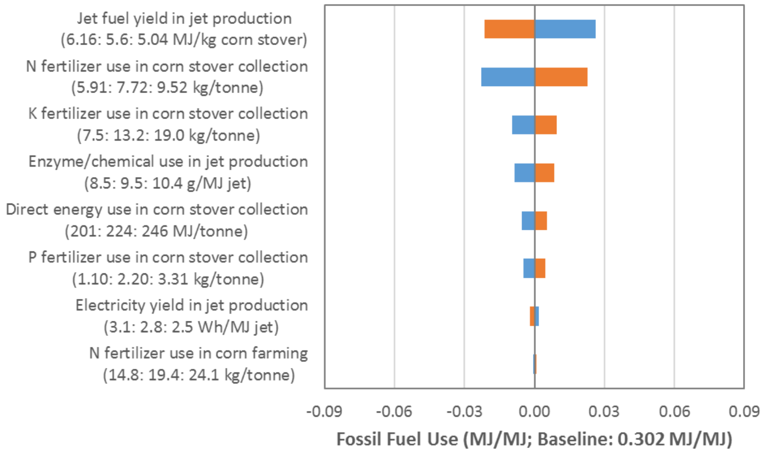

(h) Corn stover-based STJ via catalyst conversion with gasification H_2

Figure A3 Sensitivity analysis of fossil fuel use on key parameters of the ETJ and STJ pathways in MJ/MJ

Water consumption in L/MJ

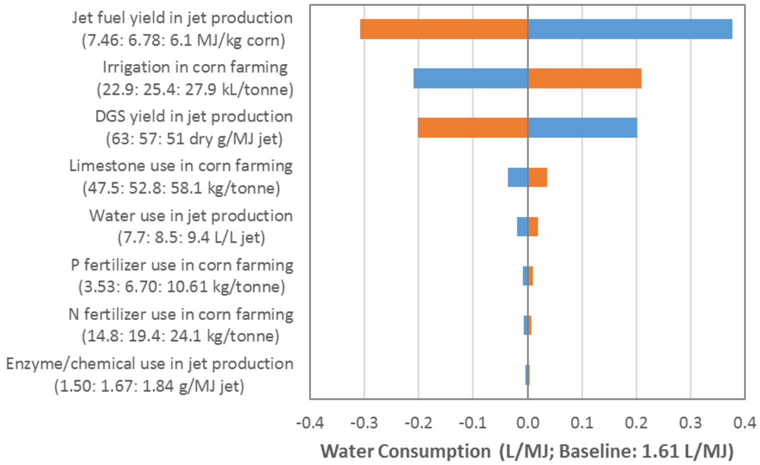

(a) Corn-based ETJ using integrated plants

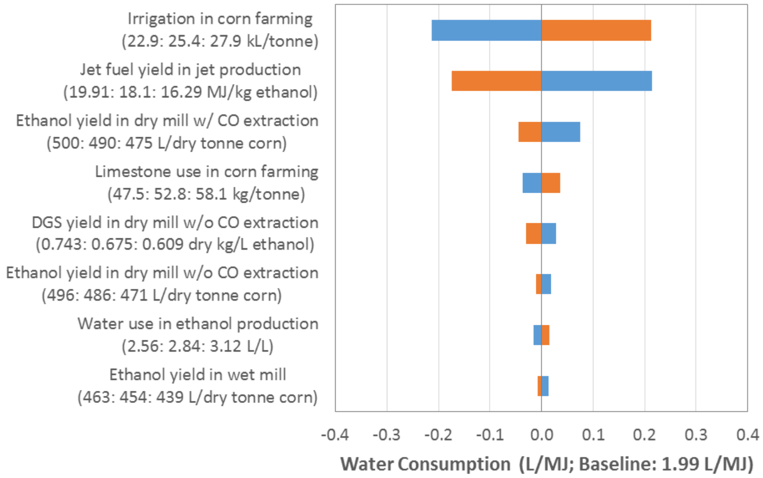

(b) Corn-based ETJ using distributed plants

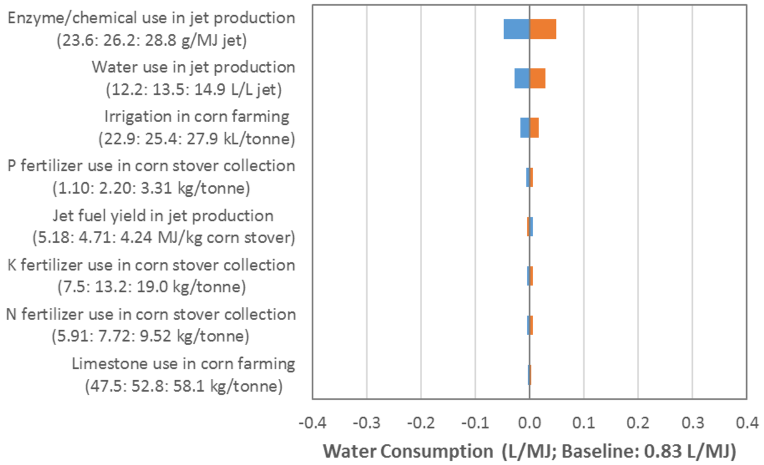

(c) Corn stover-based ETJ using integrated plants

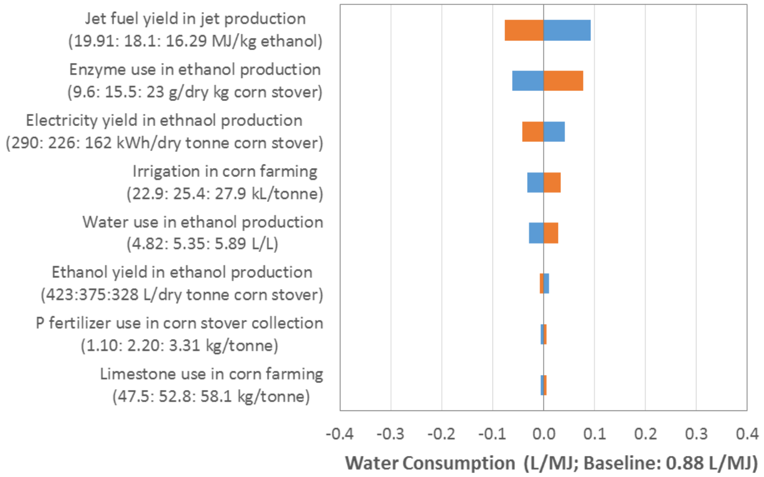

(d) Corn stover-based ETJ using distributed plants

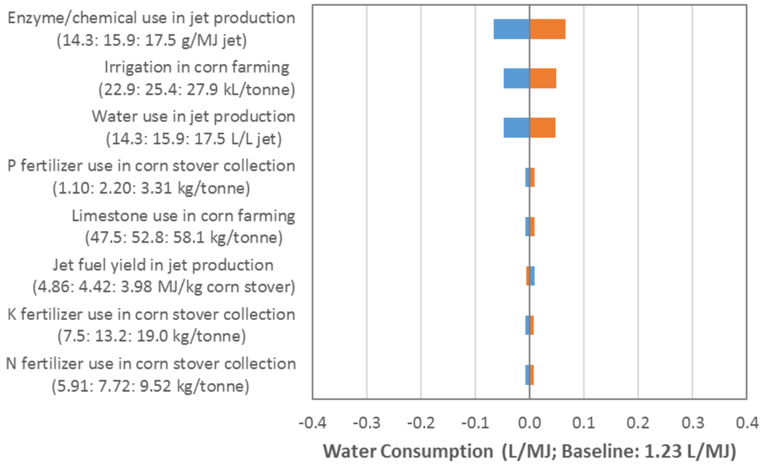

(e) Corn stover-based STJ via biological conversion

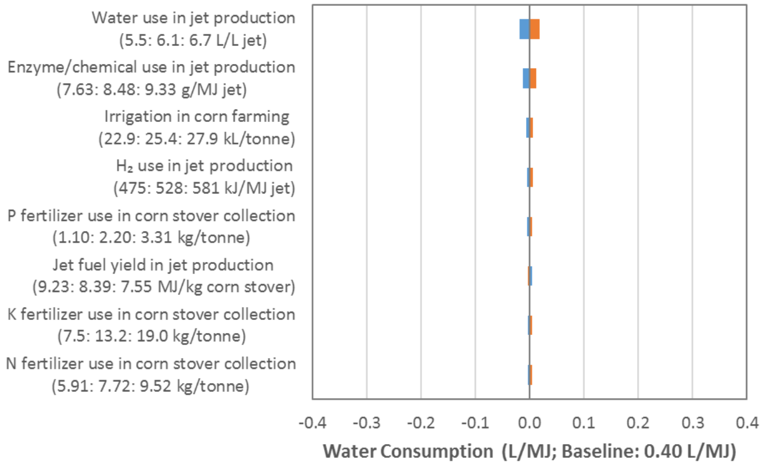

(f) Corn stover-based STJ via catalyst conversion with external H_2

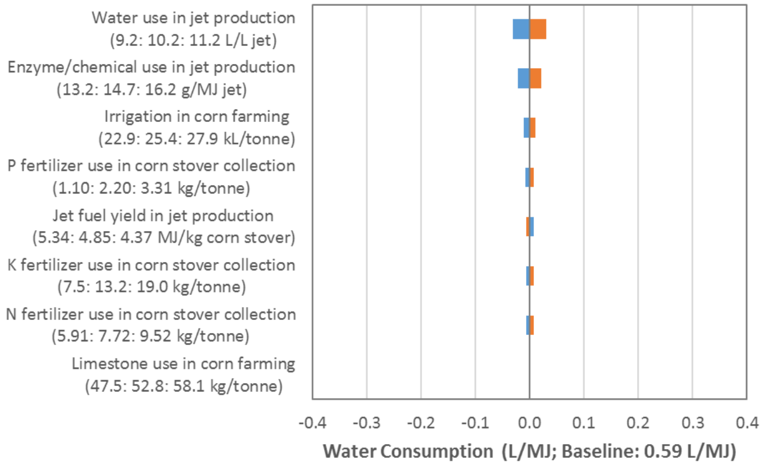

(g) Corn stover-based STJ via catalyst conversion with In-Situ H_2

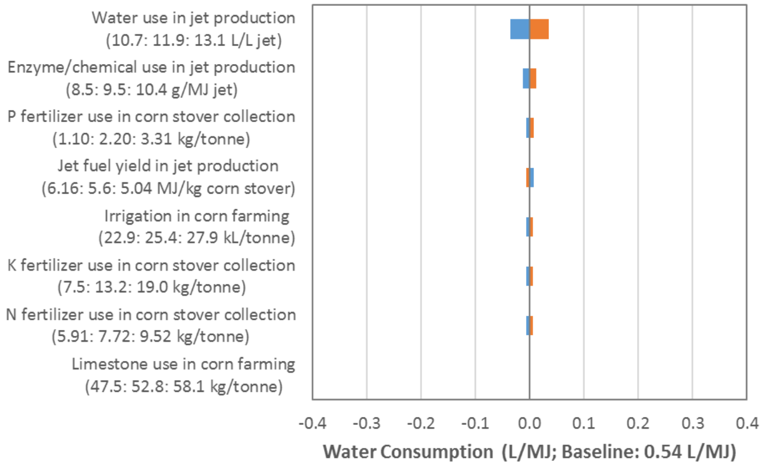

(h) Corn stover-based STJ via catalyst conversion with gasification H_2

Figure A4 Sensitivity analysis of GHG emissions on key parameters of the ETJ and STJ pathways in g CO_2e/MJ

References

1. Searchinger T, Heimlich R, Houghton RA, Dong F, Elobeid A, Fabiosa J, et al. Use of U.S. Croplands for Biofuels Increases Greenhouse Gases Through Emissions from Land-Use Change. Science. 2008;319:1238–40.

2. CARB. Proposed Regulation for Implementing Low Carbon Fuel Standards, Volume 1, Staff Report: Initial Statement of Reasons. Sacramento, CA: California Air Resources Board, California Environmental Protection Agency; 2009.

3. U.S. EPA. Renewable Fuel Standard Program (RFS2) Regulatory Impact Analysis. Washington DC: U.S. Environmental Protection Agency; 2010.

4. Hertel TW, Golub AA, Jones AD, O'Hare M, Plevin RJ, Kammen DM. Effects of US Maize Ethanol on Global Land Use and Greenhouse Gas Emissions: Estimating Market-mediated Responses. BioScience. 2010;60:223–31.

5. Tyner WE, Taheripour F, Zhuang Q, Birur D, Baldos U. Land use changes and consequent CO2 emissions due to US corn ethanol production: A comprehensive analysis [Internet]. West Lafayette, IN: Purdue University: Department of Agricultural Economics; 2010. Available from: https://www.gtap.agecon.purdue.edu/resources/download/5200.pdf

6. Laborde D. Assessing the Land Use Change Consequences of European Biofuel Policies [Internet]. Washington, DC: International Food Policy Institute; 2011. Available from: http://trade.ec.europa.eu/doclib/docs/2011/october/tradoc_148289.pdf

7. Argonne National Laboratory. Greenhouse gases, Regulated Emissions and Energy use in Transportation (GREET) Model 2013 [Internet]. 2013 [cited 2014 Apr 1]. Available from: http://greet.es.anl.gov/

8. Elliott J, Sharma B, Best N, Glotter M, Dunn JB, Foster I, et al. A Spatial Modeling Framework to Evaluate Domestic Biofuel-Induced Potential Land Use Changes and Emissions. Environ. Sci. Technol. 2014;48:2488–96.

9. CARB. Low-Carbon Fuel Standard Program [Internet]. 2015 [cited 2016 Feb 21]. Available from: http://www.arb.ca.gov/regact/2015/lcfs2015/lcfsfinalregorder.pdf

10. EU FQD. Proposal for a DIRECTIVE OF THE EUROPEAN PARLIAMENT AND OF THE COUNCIL amending Directive 98/70/EC relating to the quality of petrol and diesel fuels and amending Directive 2009/28/EC on the promotion of the use of energy from renewable sources [Internet]. Brussels, Belgium: European Commission; 2012 Oct. Report No.: COM(2012) 595. Available from: http://www.ipex.eu/IPEXL-WEB/dossier/document/COM20120595.do

11. Valin H, Peters D, van den Berg M, Frank S, Havlik P, Forsell N, et al. The land use change impact of biofuels consumed in the EU: Quantification of area and greenhouse gas impacts. Brussels, Belgium: European Commission; 2015 Aug. Report No.: BIENL13120.

12. Argonne National Laboratory. Greenhouse gases, Regulated Emissions and Energy use in Transportation (GREET) Model 2016 [Internet]. 2015 [cited 2015 Oct 10]. Available from: http://greet.es.anl.gov/

13. Wang M, Han J, Dunn JB, Cai H, Elgowainy A. Well-to-wheels energy use and greenhouse gas emissions of ethanol from corn, sugarcane and cellulosic biomass for US use. Environ. Res. Lett. 2012;7:45905.

14. Wang Z, Dunn JB, Han J, Wang MQ. Influence of corn oil recovery on life-cycle greenhouse gas emissions of corn ethanol and corn oil biodiesel. Biotechnol. Biofuels. 2015;8:178.
